# Monitoring recovery of tree diversity during tropical forest restoration: lessons from long-term trajectories of natural regeneration

**DOI:** 10.1098/rstb.2021.0069

**Published:** 2023-01-02

**Authors:** Robin L. Chazdon, Natalia Norden, Robert K. Colwell, Anne Chao

**Affiliations:** ^1^ Tropical Forest and People Research Centre, University of the Sunshine Coast, Sippy Downs, 4556 Queensland, Australia; ^2^ Department of Ecology and Evolution, University of Connecticut, Storrs, CO 06269, USA; ^3^ Programa Ciencias Básicas de la Biodiversidad, Instituto de Investigación de Recursos Biológicos Alexander von Humboldt, Bogotá, Colombia; ^4^ University of Colorado Museum of Natural History, Boulder, CO 80309, USA; ^5^ Institute of Statistics, National Tsing Hua University, Hsin Chu, Taiwan, 30043

**Keywords:** sample coverage, asymptotic species richness, standardized species diversity, evenness

## Abstract

Given the importance of species diversity as a tool for assessing recovery during forest regeneration and active restoration, robust approaches for assessing changes in tree species diversity over time are urgently needed. We assessed changes in tree species diversity during natural regeneration over 12–20 years in eight 1-ha monitoring plots in NE Costa Rica, six second-growth forests and two old-growth reference forests. We used diversity profiles to show successional trajectories in measures of observed, asymptotic and standardized tree diversity and evenness as well as sample completeness. We randomly subsampled 1-ha plot data to evaluate how well smaller spatial subsamples would have captured temporal trajectories. Annual surveys in eight 1-ha plots were missing substantial numbers of rare or infrequent species. Older second-growth sites showed consistent declines in tree diversity, whereas younger sites showed fluctuating patterns or increases. Subsample areas of 0.5 ha or greater were sufficient to infer the diversity of abundant species, but smaller subsamples failed to capture temporal trajectories of species richness and yielded positively biased estimates of evenness. In tropical forest regions with high levels of diversity, species diversity from small sample plots should be assessed using methods that incorporate abundance information and that standardize for sample coverage.

This article is part of the theme issue ‘Understanding forest landscape restoration: reinforcing scientific foundations for the UN Decade on Ecosystem Restoration’.

## 1. Introduction

Restoring tropical forests by planting trees and assisting natural regeneration is a key nature-based solution to halt and reverse biodiversity loss and mitigate climate change, which can also bring direct socio-economic benefits, such as improving water supplies, reducing erosion and enhancing crop production [1]. During forest recovery, tree communities undergo changes in forest structure [2]; shifts in dominance and species composition [3–5], phylogenetic structure [6,7], functional groups and traits [8–10]; and accumulation of rare or uncommon species [11]. The status of forest recovery is often assessed by comparing sites undergoing either natural regeneration or restoration to nearby ‘reference’ sites that represent old-growth or mature forests. Chronosequence studies examine how attributes of tree assemblages change during natural regeneration using a single measurement point in time across a group of sites within the same vicinity that have been undergoing recovery for different periods. Long-term studies, in contrast, measure changing attributes based on repeated surveys of tree abundance, size, and species composition.

Metrics of recovery of tree diversity are widely used indicators of forest restoration and recovery ‘success’ [12]. Tree diversity data are often used to compare outcomes of different restoration approaches [13,14]; assess effects of soils and climate [15]; and to detect legacies of prior land use and landscape conditions [16,17]. Assessing species diversity is also fundamental for understanding dynamic relationships between biodiversity recovery, ecosystem functions and ecosystem goods and services [18,19]. Data on recovery of tree diversity are also used to model spatial predictors of restoration potential to inform national and subnational restoration planning [20,21].

Given the importance of species diversity as a tool for assessing recovery during both forest regeneration and active restoration, robust approaches for assessing changes in tree species diversity over time are urgently needed. Chronosequence studies and meta-analyses of tropical forest succession find that tree species richness increases with plot age [22–25], whereas evenness shows no significant trend [24] or increases over time [23,25]. But comparisons of species diversity within and across study sites and regions still face major methodological challenges due to differences in plot size, stem abundance and sample completeness [26,27]. Remarkably few studies quantify changes in tree species diversity or evenness over time within individual sampling plots beyond a few years [28,29]. Longitudinal studies of tree species diversity are needed to inform an understanding of stand-level recovery dynamics that are driven by species differences in recruitment, growth and mortality rates [11,28,30,31]. Despite many advances in elucidating factors that drive forest dynamics and species demography during tropical forest regeneration [32–34], trajectories of tree diversity and evenness during tropical forest regeneration at the plot or stand level remain poorly documented and poorly understood [31,34,35].

In species-rich assemblages, such as tropical forests, small plots embedded within larger areas provide incomplete and biased information about species that occur at low relative abundance within local assemblages [36]. Small plot sizes limit the ability to make robust comparisons of tree species diversity among sites, across different age classes, or over time within a site [37]. Incomplete sampling information has the greatest impact on detecting rare or infrequent species, leading to underestimates of true species richness within assemblages and undercounting of rare species (singletons and doubletons for abundance data) [36,38]. In the most comprehensive study of recovery of tree species diversity to date—involving 56 sites (1630 plots) across tropical regions of Latin America—sample plots ranged from 0.01 to 1.0 ha, with an average of 0.09 ha [15]. Dual challenges of incomplete sampling and high levels of rarity have led to a gap in understanding of how rare or infrequent species recover during tropical forest succession. From a restoration perspective, detecting recovery of rare or infrequent species during forest restoration is a paramount objective, as these species are key indicators of conservation value and ecosystem functioning [39,40]. Detecting rare or slowly colonizing old-growth species in regenerating forests can provide strong support for protection of restored forests, including naturally regenerating areas [41].

Although the true number of species in an assemblage is unknown, it is possible to produce reliable estimates of the proportion of individuals in the assemblage that belong to undetected species based on the frequencies of species in samples [42,43]. Sample coverage is the fraction of the individuals in the assemblage (including undetected species) belonging to species represented in the sample; it is an objective measure of sample completeness at the individual level [42]. Standardizing for sample coverage or completeness controls for differences among samples in the proportion of individuals that belong to undetected species. Moreover, estimates of sample coverage can also be used to adjust the sample relative abundance of detected species to estimate true relative abundance [44]. Coverage-based approaches have recently been applied to analyses of diversity profiles of tropical stony corals, woody plant species in subtropical areas of Taiwan, spiders in montane forests of Germany, and fossil marine ostracods from Java, Indonesia [43]. Together, these analyses emphasize the importance of accounting for sample coverage when comparing samples from different sites, regions or geological periods.

Here, we exploit a unique long-term dataset to conduct a detailed and statistically robust analysis of trajectories of tree species diversity during natural regeneration in six tropical rainforest plots in NE Costa Rica, beginning 12–25 years after pasture abandonment. We examine temporal trajectories over 12–20 years to estimate sample completeness (coverage) and assemblage species diversity of trees (stems ≥5 cm dbh), based on annual sampling of 1-ha plots. Our approach, using standardized measures of species diversity based on sample coverage, is the first to examine long-term successional trajectories of tree species diversity and evenness in tropical secondary forests in comparison with nearby old-growth (reference) forests. We use diversity profiles based on Hill numbers to examine successional trajectories in observed and estimated tree diversity and evenness [42,43]. Whereas observed data on species richness and abundance describe the sample plot, estimates of species diversity provide information about the assemblage from which the plot is assumed to be a representative sample. Our approach provides insights into the contributions of differences in species relative abundance and evenness to estimates of assemblage diversity and sample completeness. Evenness is often viewed as a proxy for species dominance, but it is also a highly sensitive indicator of rare or infrequent species in samples. We adopt a four-step approach to comparing species diversity and evenness across assemblages based on incomplete sample data, linking sample completeness, diversity estimation, rarefaction and extrapolation and evenness [43]. Further, we use data from six 1ha second-growth plots to evaluate how smaller spatial subsamples (0.1 ha, 0.2 ha, 0.5 ha and 0.8 ha) reveal (or fail to reveal) diversity and evenness trajectories and plot differences.

Our study addresses three main questions:

(1) How effective are long-term monitoring plots of 1 ha for capturing complete information about species diversity within tree assemblages?
(2) What are the best measures of species diversity for assessing change over time (12–20 years) in six secondary forests and for comparison in two old-growth ‘reference forests’?
(3) How are estimates of observed, asymptotic and standardized species diversity, sample completeness and standardized evenness affected by sampling effort?

These findings provide insights into how to monitor changes in tree species diversity in assemblages using incomplete sample plot data from ecological restoration projects or naturally regenerating forests. We conclude with recommendations for monitoring tree species diversity during restoration and natural regeneration of tropical forests.

## 2. Methods

### (a) Vegetation sampling and database management

We monitored tree species abundance annually in two old-growth and six second-growth rainforest sites in northeastern Costa Rica (table 1). In each site, a 1-ha permanent plot (50 m × 200 m) was established in 1997 or 2005 in which all trees were uniquely tagged and identified. We surveyed species abundance for all stems with DBH of 5 cm or more each year from 1997 to 2017 for four second-growth forests (CR, LEP, TIR and LSUR), and from 2005 to 2017 for two primary forests and the two youngest second-growth forests (FEB and JE). Each year, new recruits and dead individuals were recorded and the diameters of tagged trees were measured at breast height or above buttresses or stem irregularities to the nearest 0.1 mm with a nylon diameter tape. Measurement points were marked with paint on trees to reduce errors in diameter increment measurements. Species were identified in the field by expert local parataxonomists, and identifications were confirmed by collecting voucher specimens and comparing with identified herbarium specimens at the La Selva Biological Station Herbarium and the Costa Rican National Herbarium. A summary of statistics for the eight sites is provided in electronic supplementary material, appendix S1 (electronic supplemental material, data).

**Table 1.** Site names and ages in 2017. Sites are located within Sarapiquí County in northeastern Costa Rica.

site code	site name	age in 2017 (years)	age at first census (years)	year of first census	location	mean plot elevation (m)	latitude/longitude of plot origin
JE	Juan Enriquez	22	10	2005	Chilamate	129	N10 27.266
							W84 03.935
FEB	Finca El Bejuco	22	10	2005	Chilamate	106	N10 27.267
							W84 03.921
LSUR	Lindero Sur	32	12	1997	La Selva	144	N10 24.790
							W84 01.675
TIR	Tirimbina	35	15	1997	La Virgen	217	N10 24.161
							W84 06.700
LEP	Lindero El Peje	40	20	1997	La Selva	98	N10 25.885
							W84 02.029
CR	Cuatro Rios	45	25	1997	La Virgen	230	N10 23.392
							W84 07.701
LEP primary	Lindero El Peje Primary	>200	>200	2005	La Selva	132	N10 25.438
							W84 02.357
SV primary	Selva Verde Primary	>200	>200	2005	Chilamate	140	N10 26.442
							W84 03.997

### (b) Analysis of sample completeness

Before comparing diversity across space or over time, we first quantified sample completeness for each annual survey [43]. For the time series data within each site, we focus on sample completeness of orders *q* = 0, 1, and 2 computed for each year. When *q* = 0, only species incidence (presence) is considered, and completeness reduces to the proportional contribution of observed species to the estimated true (asymptotic) richness (observed plus undetected), based on the Chao1 estimator. This measure expresses the conventional sense of sample completeness, in which all *species* are treated equally and given equal weight. Sample completeness of order *q* = 1 reduces to sample coverage, the fraction of an assemblage's individuals that belong to the observed species [45,46]. For this measure, all *individuals* are treated equally and given equal weight. Thus, each *species* is given a weight proportional to its abundance. For sample completeness of order *q* = 2, each species is given a weight proportional to the square of its abundance, making this measure disproportionally sensitive to abundant species.

### (c) Asymptotic and non-asymptotic diversity analyses of 1-ha plots

We applied the interpolation and extrapolation standardization procedure of [26] and [42], as implemented in the software iNEXT-4-steps (https://chao.shinyapps.io/iNEXT_4steps/) [43], to analyse the yearly tree species abundance data. We adopted the asymptotic estimates developed by Chao and Jost [47] for Hill numbers of orders *q* = 0, 1 and 2. For *q* = 0, their formula reduces to the Chao1 richness estimator. Hill numbers for order *q* ≥ 0 are all in units of ‘species’ or ‘species equivalents’ and include the three most widely used species diversity measures (species richness, exponential Shannon diversity and inverse Simpson diversity) as special cases, of orders *q* = 0, 1 and 2, respectively. A bootstrap method was applied to obtain the associated confidence intervals [42]. In our yearly samples, none of the size-based rarefaction and extrapolation sampling curves stabilized for species richness (*q* = 0; see electronic supplementary material, appendix S2 in supplemental data), even when observed sample size was doubled by statistical extrapolation. Thus, our asymptotic species richness estimate represents only a lower bound and exhibits negative bias (underestimation). Consequently, the difference between the asymptotic estimate and observed richness (the number of species observed in each plot in each year) represents the minimum number of undetected species. Rarefaction and extrapolation sampling curves for *q* = 1 and *q* = 2 do stabilize, meaning the asymptotic estimates of *q* = 1 and *q* = 2 are reliable (i.e. data are sufficient to infer the true diversity of *q* = 1 and *q* = 2, but not *q* = 0; electronic supplementary material, appendix S2 in supplemental data).

Data from 1-ha plots do not contain sufficient information to infer the richness of entire assemblages due to insufficient information on rare species that are present in the surrounding forest but absent, by chance, by the plot. Therefore, for species richness, we compared *non-asymptotic* diversity estimates based on a standardized value of sample coverage. Chao and Jost [42] proposed a coverage-based methodology for species richness by standardizing all samples to the same level of sample coverage (i.e. comparing species richness for a standardized fraction of an assemblage's—not the plot's—individuals). The concept and methodology were subsequently extended to Hill numbers by Chao, Chiu and Jost [48]. We used iNEXT (as extended to iNEXT4Steps) software to facilitate all computation and graphics [49]. In the coverage-based rarefaction and extrapolation of sampling curves, we standardized by the maximum coverage value possible (*C*_max_), i.e. the minimum among the coverage values for samples extrapolated to double the observed sample size—‘max’ because it is the highest level of coverage we can use for comparison. A maximum of doubling by extrapolation is a rule of thumb, to avoid excessive uncertainty. To avoid discarding useful data, all yearly samples within a site were first extrapolated to double their observed sample sizes [42]. We then computed the minimum among the coverage values obtained from those extrapolated samples. Because the minimum coverage value among the extrapolated (doubled) samples for the eight sites was 96.52%, we defined *C*_max_ = 96.52% and used this coverage value to standardize species diversity measures.

### (d) Coverage-based evenness

Evenness is a measure of species variation in relative abundance. When species abundances are perfectly even, diversity of all orders *q* ≥ 0 are identical and equal to species richness. When species abundances are not even, species diversity declines as the diversity order *q* increases. For *q* = 0, it is not meaningful to evaluate evenness, as species abundances are disregarded. The magnitude of the *difference* between diversity of order *q* > 0 and species richness (*q* = 0) reflects the extent of unevenness among species abundances. Chao and Ricotta [50] estimated evenness as the slope connecting two points with diversity orders 0 and any *q* > 0 in the Hill-number profile. The slope is then normalized to the range of [0,1], where minimum evenness is 0 and maximum evenness is 1. Because richness is involved in the resulting formula, evenness can be evaluated only at a standardized level of sample coverage. Here, we focus on evenness measures of orders *q* = 1 and *q* = 2 at the coverage level of *C*_max_ = 96.52%.

### (e) Diversity analysis by subsampling six second-growth forests

To evaluate how estimates of observed, asymptotic, and standardized species diversity are affected by sampling effort, we took advantage of the fact that each of the plots considered in our diversity analyses is 1 ha, divided into 100 subplots of 10 m × 10 m. Since many studies of regenerating forests are based on plots that are less than 1 ha, we randomly selected sub-areas from 1 ha to examine the sensitivity of estimates of diversity and evenness measures to the size of subsampled area. For six second-growth forests (CR, LEP, TIR, LSUR, FEB, JE) we conducted simulations for four sub-sampling schemes, randomly selecting 10 subplots (0.1 ha), 20 subplots (0.2 ha), 50 subplots (0.5 ha) and 80 subplots (0.8 ha), without replacement, and repeated our diversity analyses for these sets of subplots. To minimize sampling variability, we generated the subsamples 200 times and obtained the mean values. We present results as mean values of 200 simulated subsamples.

## 3. Results

### (a) How effective are long-term monitoring plots of 1 ha for capturing complete information about species diversity within assemblages?

**Figure 1.**
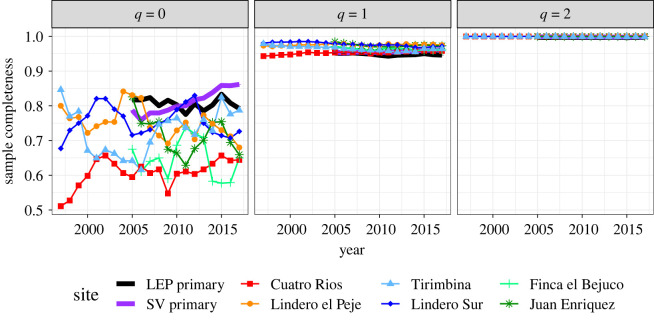
Temporal pattern of sample completeness by site and census year. Sample completeness for order *q* = 0 is lower and less stable in second-growth sites than old-growth sites. Site abbreviations are explained in table 1. The plots of estimated sample completeness curves for orders *q* = 0 (left panel), *q* = 1 (middle panel) and *q* = 2 (right panel) are based on yearly species abundance data for eight forest sites from 1997 (or 2005) to 2017; a summary of the data appears in electronic supplementary material, appendix S1. The sample completeness of order *q* = 0 represents an *upper bound* for the proportion of species that were observed in the sample; its complement represents a minimum proportion of undetected species. The sample completeness of order *q* = 1 represents sample coverage (the fraction of an assemblage's individuals that belong to the observed species). The sample completeness of *q* = 2 is disproportionally sensitive to abundant species.

Annual surveys for the 1-ha plots revealed virtually all of the common or abundant species from their respective assemblages, but these surveys were missing significant numbers of rare (uncommon everywhere) or infrequent (patchy distributions, locally rare but more common nearby) species [51]. In old-growth forests, at least 20% of the species in the entire assemblage were not detected in annual plot inventories. Sample completeness for species richness (*q* = 0) for old-growth plots was stable over time and was generally higher (with average values about 80% over time) compared to the second-growth plots (figure 1).

Second-growth forests showed non-uniform trends in sample completeness for species richness over time and across site ages; completeness fluctuated greatly, and the average completeness values across time were below 80%, especially for the Cuatro Rios site, which has the highest proportion of singletons across all sites (figure 1, electronic supplementary material, appendix S1). Sample completeness based on species abundance (*q* = 1) exceeded 95% in nearly all yearly samples and was steady over time, meaning that at most 5% of each assemblage's individuals belonged to the undetected species. For abundant species (*q* = 2), all yearly data provided nearly complete samples of their respective assemblages, indicating that all abundant species were detected in each yearly census.

### (b) What are the best measures of species diversity for assessing change over time in six secondary forests and for comparison with ‘reference’ old-growth forests?

**Figure 2.**
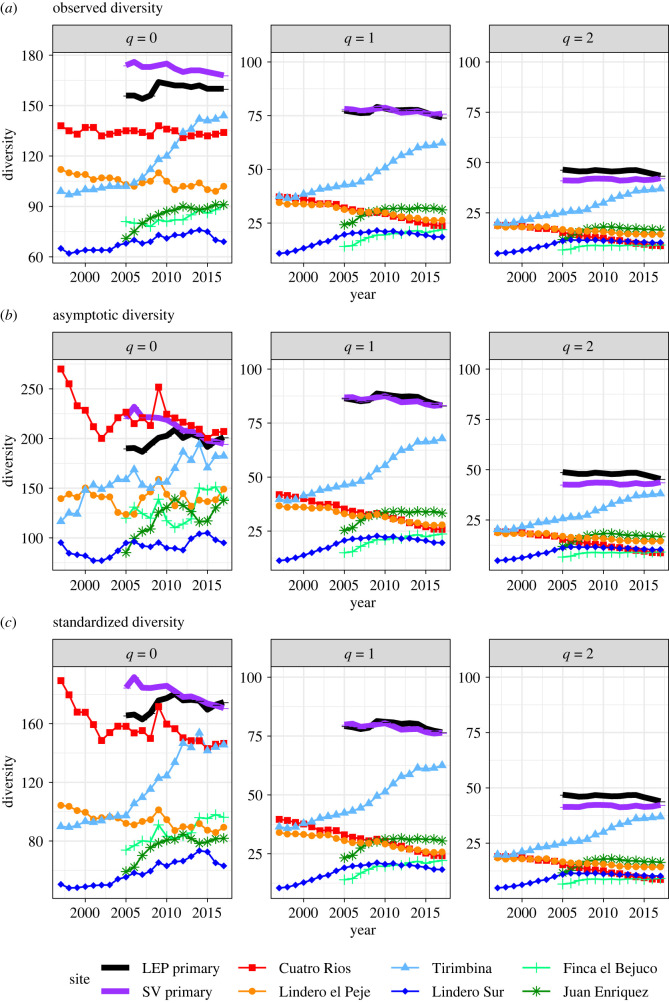
Diversity patterns across eight sites based on different estimation methods. Tree diversity measures increase over time in young second-growth sites but decrease over time in older second-growth sites. Site abbreviations are explained in table 1. Temporal diversity patterns are based on yearly tree species abundance data using three different estimation methods: (*a*) observed diversity; (*b*) asymptotic diversity estimates; and (*c*) standardized diversity estimates under a maximum coverage value of *C*_max_ = 96.52% (i.e. the minimum among the coverage values for samples extrapolated to double the observed sample size). The three sub-panels within each panel represent, respectively, the diversity of order *q* = 0 (sub-panel 1), *q* = 1 (sub-panel 2) and *q* = 2 (sub-panel 3). Note the scale difference in *Y*-axis among the three panels.

For common and abundant species (*q* = 1 and *q* = 2), the observed, asymptotic and standardized diversity estimates exhibit identical trajectories (figure 2*a–c*). The two reference forests consistently showed higher tree species diversity of common and abundant species and higher evenness than the second-growth forests (electronic supplementary material, appendix S3 in supplemental data). The two older second-growth sites, CR and LEP, showed consistent decreases in tree diversity, whereas the younger sites showed more-fluctuating patterns or strong (TIR) or modest increases (LSUR, FEB, JE). Diversity measures in 2017 for common (*q* = 1) and abundant (*q* = 2) species did not correspond with plot age, as the TIR plot (35 years) showed the highest observed, asymptotic and coverage-based species diversity, whereas LSUR (32 years) and JE (22 years) showed the lowest species diversity measures. A replot of figure 2 for the six second-growth forests with respect to age is provided in electronic supplementary material, appendix S2 (supplemental data).

Species diversity measures based solely on species richness (*q* = 0) varied greatly across observed, asymptotic and standardized measures. Based on observed (plot) data (figure 2*a*), tree species richness was lower for the six second-growth forests compared to the reference forests (*q* = 0), but for asymptotic richness, the CR site had higher levels than the reference forests, due to the high number of singletons in this plot (figure 2*b*; Appendix S1 in supplemental data). The substantial difference between observed (figure 2*a*) and asymptotic species richness estimates (figure 2*b*) shows that rare species remained undetected in each yearly sample (figure 1).

Given the extent of undetected rare species, diversity comparisons of species richness should be based on coverage-based standardized diversity (figure 2*c*). Standardized species richness estimates of the two reference forests were higher than second-growth forests. The two older second-growth forests (CR and LEP) generally exhibited a decreasing trend in richness with time, whereas the four younger forests generally showed an increasing trend (figure 2, panel *c*). When comparing species richness patterns (*q* = 0), standardizing for sample coverage greatly improved the rigor of spatial or temporal comparisons, by accounting for differences in sample completeness. These results show that standardized species richness of second-growth forests in 2017 was highest in CR and TIR and lowest in LSUR, and was not related to stand age.

### (c) Effects of sampling effort on estimates of sample completeness and diversity measures

**Figure 3.**
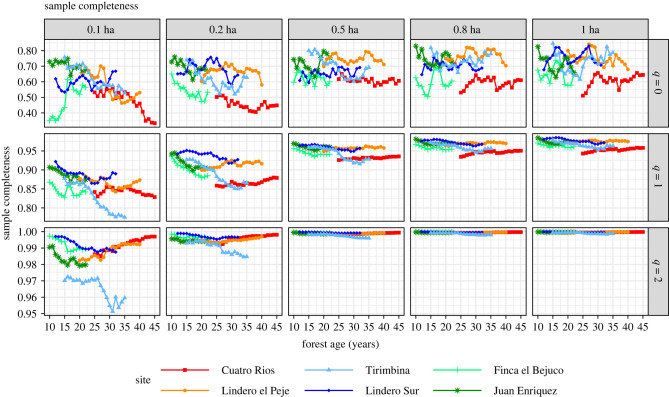
Sample completeness of orders *q* = 0 (row 1), *q* = 1 (row 2) and *q* = 2 (row 3) when 10 subplots (0.1 ha, column 1), 20 subplots (0.2 ha, column 2), 50 subplots (0.5 ha, column 3) and 80 subplots (0.8 ha, column 4) were randomly selected (without replacement) from each of the six second-growth forests. Sample completeness increases with sample size. Site abbreviations are explained in table 1. The curves for each subsampled area represent average values over 200 simulation trials. Note the scale difference in *Y*-axis among three rows.

Generally, estimated sample completeness demonstrated a monotonically increasing pattern as sample effort increased (figure 3). Sample coverage varied from 80% to 99% with increases in plot size from 0.1 ha to 1 ha (figure 3). For abundant species (*q* = 2), once subsampled area exceeded 0.5 ha, nearly all sample completeness values approached unity (complete coverage), except for the TIR site.

**Figure 4.**
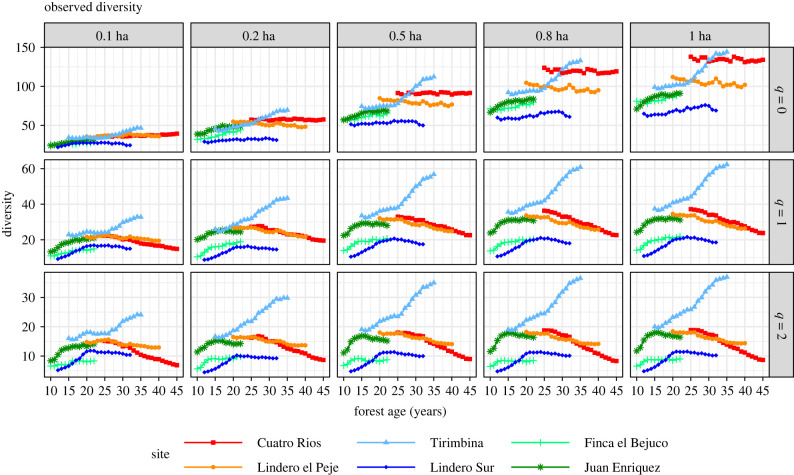
Observed diversity for orders *q* = 0 (row 1), *q* = 1 (row 2) and *q* = 2 (row 3) when 10 subplots (0.1 ha, column 1), 20 subplots (0.2 ha, column 2), 50 subplots (0.5 ha, column 3) and 80 subplots (0.8 ha, column 4) were randomly selected (without replacement) from each of the six second-growth forests. Observed tree diversity increases dramatically with sample size, especially for *q* = 0. Site abbreviations are explained in table 1. The curves for each subsampled area represent the average values over 200 simulation trials. Note the scale difference in *Y*-axis among three rows.

**Figure 5.**
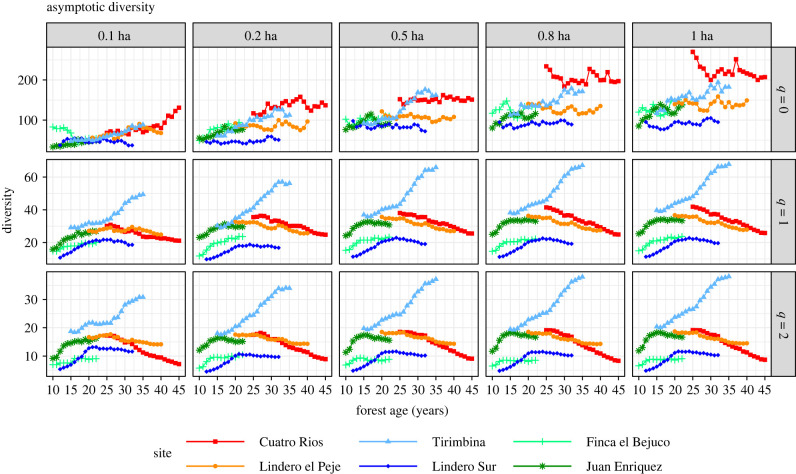
Estimated asymptotic diversity. The asymptotic diversity estimates of orders *q* = 0 (row 1), *q* = 1 (row 2) and *q* = 2 (row 3) when 10 subplots (0.1 ha, column 1), 20 subplots (0.2 ha, column 2), 50 subplots (0.5 ha, column 3) and 80 subplots (0.8 ha, column 4) were randomly selected from each of the six second-growth forests. Asymptotic tree diversity increases dramatically with sample size, especially for *q* = 0. Site abbreviations are explained in table 1. The curves for each subsampled area represent the average values over 200 trials. Note the scale difference in *Y*-axis among three rows. (In electronic supplementary material, appendix S4, the same figure with 95% confidence intervals based on 200 bootstrap replications is provided).

When subsample area was increased from 0.1 ha to 1 ha, all the observed diversity measures increased, regardless of diversity order *q* (figure 4). The increment was substantial for rare species (as reflected by the curves for *q* = 0 in row 1), moderate for common species (*q* = 1), and small for abundant species (*q* = 2). Temporal declines in species richness (*q* = 0) in the two oldest second-growth sites were not detected for subsample areas of less than 0.5 ha. Asymptotic diversity measures for *q* = 0 (species richness) also increased dramatically with sample plot size (figure 5). Based on 1-ha plots, estimated assemblage richness in second-growth sites generally exceeded 100 tree species and exceeded even 200 species in the Cuatro Rios site. If only 0.1-ha sub-plots were sampled, however, estimated assemblage species richness estimates were generally below 100 (figure 5). The rank order of assemblage species richness among plots remained consistent across sample effort but shifted with sample effort when abundance data were included.

**Figure 6.**
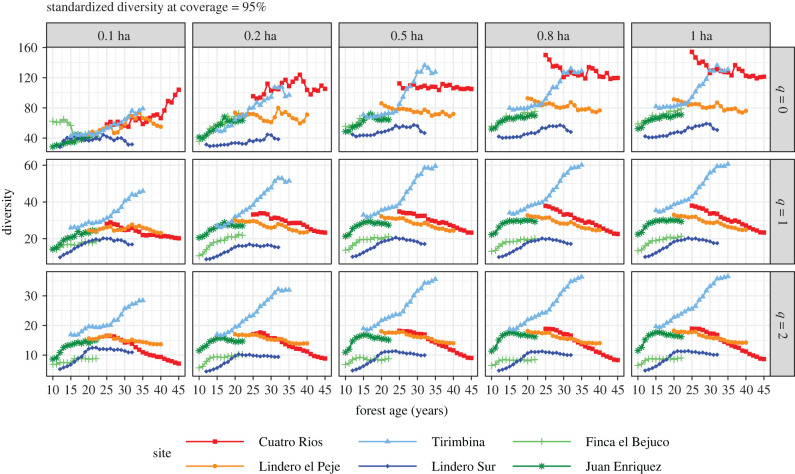
Rarefied/extrapolated diversity for sample coverage of 95%. The rarefied/extrapolated diversity of orders *q* = 0 (row 1), *q* = 1 (row 2) and *q* = 2 (row 3) when 10 subplots (0.1 ha, column 1), 20 subplots (0.2 ha, column 2), 50 subplots (0.5 ha, column 3) and 80 subplots (0.8 ha, column 4) were randomly selected, without replacement, from each of the six second-growth forests. Standardized tree diversity shows consistent temporal patterns with increasing sample size, especially for *q* = 0. Site abbreviations are explained in table 1. All data in each site were either rarefied or extrapolated to a common coverage value 95%. The curves for subsampled areas represent the average values over 200 trials. Note the scale difference in *Y*-axis among the three rows. (In electronic supplementary material, appendix S4, the same figure with 95% confidence intervals based on 200 bootstrap replications is provided).

For estimates of species richness, none of the subsampled areas provides results comparable to those based on the full 1 ha (figure 6). Standardized estimates of assemblage species richness increased with sample size from 0.1 to 0.8 ha, and then stabilized up to 1 ha (*q* = 0; figure 6). For common and abundant species (*q* = 1 and *q* = 2), the decline of species diversity over time in the older sites is clearly evident for all subsampled areas, whereas species diversity initially increased and then levelled off or declined slightly in the three youngest sites. For *q* = 1 and *q* = 2, standardized diversity patterns/estimates (figure 6) closely resemble those of asymptotic diversity (figure 5). These results show that subsample plots of 0.5 ha are sufficient to infer the diversity of common and abundant species.

### (d) Effects of plot size on successional trajectories of standardized evenness

**Figure 7.**
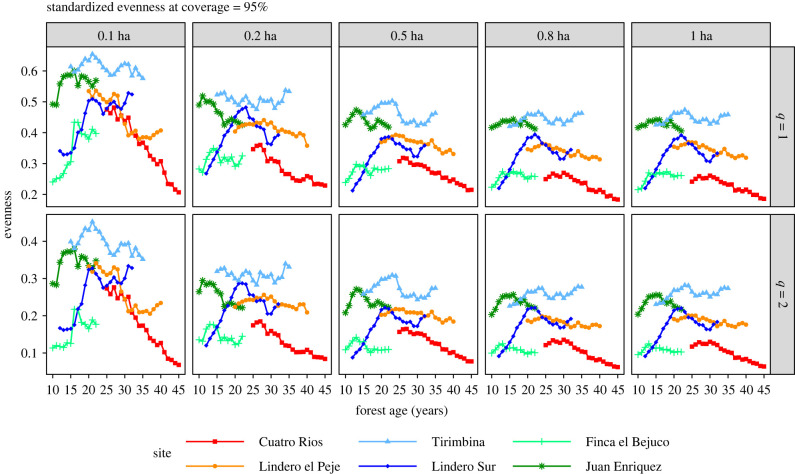
The estimated evenness for orders *q* = 1 (upper row) and *q* = 2 (lower row) under the coverage value of 95% when 10 subplots (0.1 ha, column 1), 20 subplots (0.2 ha, column 2), 50 subplots (0.5 ha, column 3) and 80 subplots (0.8 ha, column 4) were randomly selected, without replacement, from each of the six second-growth forests. Measures of standardized evenness decrease as sample sizes increase. Site abbreviations are explained in table 1. The curves for subsampled areas represent the average values over 200 trials. Note the scale difference in *Y*-axis among the three rows.

Standardized evenness (based on coverage of 95%) decreased as subsampled area increased (figure 7). The patterns were similar for a standardized coverage value of 90% (electronic supplementary material, appendix S5 in supplemental data). This decrease appears because a small sub-plot with 0.1 ha or 0.2 ha tends to include common and abundant species but few rare species, leading to higher measures of evenness. Measures of evenness from subsample plots below 0.5 ha were heavily positively biased for evenness, compared to 1-ha plots. Evenness patterns were consistent for both diversity orders, although the values for *q* = 2 are much lower than for *q* = 1. As with standardized diversity measures, subsample plot areas of 0.5 ha and 0.8 ha yielded inferences on species evenness similar to estimates based on 1 ha samples. The TIR site showed the highest evenness, with little change over time. The two oldest second-growth sites, LEP and CR, showed declines in evenness over time, with CR showing the lowest evenness values. The two youngest sites (FEB, JE) showed initial increases in evenness followed by stabilization or declines. LSUR showed a sustained increase in evenness for 8 years, followed by a decline and a later increase after 18 years.

## 4. Discussion

Our finding that sample plots of 1 ha miss a significant number of tree species that occur within local assemblages frames the principal challenge in assessing tree diversity changes over time and comparing recovering forest sites to reference sites. Observed species richness is always a negatively biased measure of true assemblage richness, to varying degrees, such that comparisons among plots may not reflect actual differences in assemblage diversity [38]. Our results show that standardizing species diversity and evenness as a function of statistical sample coverage provides the most robust basis for comparisons across plots and within plots over time [42]. Comparison of species richness should be based on estimates that consider a fixed fraction of the true, regional assemblages' individuals, i.e. a fixed value of sample coverage. The use of standardized diversity measures improved comparisons for small subsamples between 0.1 and 0.5 ha that are commonly used in studies of forest recovery and restoration [15,52] (figure 6). Still, plots smaller than 0.5 ha may be insufficient to offer accurate estimations of species diversity. For species diversity measures based on common or abundant species, 1-ha plots sampled virtually all of the species from their respective assemblages. Subsamples of 0.5 and 0.8 ha also showed very high coverage for abundant species (figure 3). We therefore recommend that restoration projects in tropical forest regions that aim to restore native biodiversity design monitoring plans based on sample areas of at least 0.5 ha, and focus on common or abundant species, which are less sensitive to sampling effort. Although rare species are the most vulnerable to being lost and may support critical ecosystem functions [40], common and abundant species may be better indicators for recovery, as they contribute to convergence with old-growth reference forests [3,11,53].

Changes in species diversity in recovering forests, however, do not show steady increases over time until reaching a plateau, which is the typical pattern portrayed in conceptual frameworks for succession. Rather, we found divergent and fluctuating patterns of tree species diversity during the first 10–50 years of succession. For instance, standardized diversity measures (figure 2) showed a clear divergence in temporal trajectories between the two oldest and the two youngest second-growth forests. When viewed in terms of stand age, however, these trends suggest that species diversity may reach an initial peak at around 20–30 years within our study region (electronic supplementary material, appendix S3 in supplemental data). Tirimbina is the only second-growth forest that showed consistent increases in species diversity during this time interval, when forests are transitioning from the stand-thinning phase to the understory reinitiation phase [54]. These trends suggest that recovery of species richness during forest regeneration may show periods of increases and decreases that correspond to a shift in dominance from pioneer species to shade-tolerant species [55]. Standardized evenness showed increasing trends in young sites and decreasing trends in older sites (figure 7) that may be associated with successional transition from the thinning phase to the understory re-initiation phase [54,55]. The temporal trends revealed by our data should be examined across a larger set of study plots undergoing natural regeneration or restoration. Overall, these findings suggest that consistent increases in species richness and diversity over time may not be an appropriate criterion for restoration success. Particularly during the first 20–50 years of stand development, dramatic internal dynamics such as stand thinning, shifts in species dominance and understory recruitment may cause species richness and diversity to fluctuate. In addition to these changes, mostly driven by shifts in the ecological processes underlying succession, successional trajectories may be quite idiosyncratic, even when controlling for the multiple factors that cause such variation, such as land use, priority effects, initial conditions and environmental heterogeneity [31]. Moreover, variability in landscape spatial configuration amplifies the unpredictability of succession [34]. This effect holds particularly true for rare species, which show greater fluctuations over time. Thus, indicators based on common (*q* = 1) or abundant species (*q* = 2) provide a more reliable assessment of overall recovery of tree species diversity in restoration projects.

The old-growth (reference) forests in our study had higher mean sample coverage, higher observed species diversity and higher standardized diversity across all three diversity orders compared to 12–45 year old second-growth forests (figures 1 and 2). This result is not surprising, as old-growth forests have had centuries to accumulate species and to reach a stable relative abundance structure. Based on our ‘long-term’ study of 20 years, we captured trajectories that apply to a short time interval compared to the many decades or centuries of species accumulation required to achieve the high levels of species richness and evenness in reference forests. Our findings highlight the importance of having one or more reference sites to evaluate recovery of species diversity [3,15] as compositional variation is high in tropical forests as a result of both environmental heterogeneity and dispersal limitation [56]. Older successional forests may provide useful reference sites in cases where old-growth forests are no longer present, but the temporal stability of species diversity and evenness should also be examined in these older sites. Older successional forests in tropical regions are likely to be missing species that accumulate over decades or centuries in old-growth forests.

Our study provides several lessons for monitoring and enhancing tree diversity during restoration projects based on assisted regeneration or tree planting to accelerate recovery of forest biodiversity. First, diversity measures used as indicators of recovery should focus on common or abundant species. Even censuses based on sample areas of 1 ha or less will miss a significant fraction of rare species that are actually present in the assemblage. Including small size classes in sampling methods may increase the likelihood of detecting increased numbers of species of recent recruits [57]. Under such a scenario, plot size is an important point to consider. From a statistical perspective, few larger plots are useful to evaluate species spatial aggregation or association/dissociation in a fixed area. By contrast, many small plots allow better assessment of species diversity across a landscape because this sampling method provides a more representative sample of the species pool occurring in the whole area of interest. If treatment or experimental areas are small and completely sampled, these data include the entire assemblage, eliminating the need for statistical sampling approaches.

Second, when information on recovery of rare species or species of conservation interest is needed, plot-based sampling may not be a suitable approach. Covering larger areas using transects or targeted searches for particular species may provide more useful information than using smaller, completely sampled plots [58]. Alternatively, stratified cluster sampling across heterogeneous edaphic conditions or environmental gradients in regenerating forest areas may enable more effective sampling of rare or infrequent species, compared to one sample plot of the same total area [37].

Third, maximizing recovery of species diversity may require silvicultural interventions within plots to reduce abundance of dominant species, control the spread of invasive species, or to increase microhabitat heterogeneity [59,60]. Enrichment planting of locally adapted tree species (often accompanied by silvicultural treatments) can also be a useful approach to boost site-level species richness and diversity [60–62].

Finally, trees are only one component of the biodiversity in regenerating forests of the tropics, where over 90% of woody species have animal-dispersed seeds and nearly 70% are pollinated by animals [63]. Inventories focused on sampling a range of taxonomic groups comprising flora and fauna will provide a more complete picture of the recovery of biodiversity in restored and regenerating forests [13].

## Data Availability

All data used in this study are available from the Dryad Digital Repository: https://doi.org/10.5061/dryad.ncjsxksvr [64]. The data are provided in electronic supplementary material [65].
